# Management Outcomes in Males With Hypogonadotropic Hypogonadism Treated With Gonadotropins

**DOI:** 10.7759/cureus.35601

**Published:** 2023-02-28

**Authors:** Bahaa O Sahib, Ibrahim H Hussein, Nassar T Alibrahim, Abbas A Mansour

**Affiliations:** 1 Diabetes and Endocrinology, Fiaha Specialized Diabetes, Endocrine and Metabolism Center (FDEMC) University of Basrah, Basrah, IRQ; 2 Endocrinology, Faiha Specialized Diabetes, Endocrine and Metabolism Center (FDEMC) College of Medicine, University of Basrah, Basrah, IRQ; 3 Diabetes and Endocrinology, Faiha Specialized Diabetes, Endocrine and Metabolism Center (FDEMC) College of Medicine, University of Basrah, Basrah, IRQ

**Keywords:** gonadotropins, hypogonadotropic hypogonadism, iraq, male hypogonadism, spermatogenesis

## Abstract

Background

Hypogonadotropic hypogonadism is an important cause of male infertility and loss of secondary sexual characteristics. Gonadotropin replacement is mandatory for sexual function, bone health, and normal psychological status. This study is to compare the effectiveness of different gonadotropin therapy modalities in the management of male hypogonadism.

Methods

A randomized open-label prospective study of 51 patients attended the Faiha Specialized Diabetes, Endocrine and Metabolism Center (FDEMC) with hypogonadotropic hypogonadism, divided randomly into three groups. The first group was treated with human chorionic gonadotropin (hCG) alone, the second group was treated with a combination of both hCG and human menopausal gonadotropin (HMG), while the third group started with hCG alone then followed by combination therapy after six months.

Results

All modalities of therapy result in a significant increase in mean testicular volume although no clinically significant difference between the groups, but the combination group had the highest increment. The increment in serum testosterone level was statistically significant among the different groups of treatment (p-value < 0.0001). When comparing groups, a higher mean maximum testosterone level (710.4±102.7 ng/dL) was obtained with the combination group followed by the sequential group, with mean maximum testosterone levels (636.0±68.6 ng/dL) (p-value = 0.031). Factors negatively affecting testosterone level include BMI > 30 kg/m^2^, initial testicular volume < 5 mL, and duration of therapy < 13 months.

Conclusions

Induction of puberty using recombinant hCG alone is sufficient to induce secondary sexual characteristics, while for fertility issues combination from the start or sequential therapy has better for spermatogenesis. There was no effect of prior exogenous testosterone treatment on final spermatogenesis.

## Introduction

Hypogonadotropic hypogonadism (HH) is defined as failure of gonadotropin production secondary to pituitary or hypothalamic cause that results in low circulating testosterone with symptoms and signs of androgen deficiency [1]. The prevalence of male hypogonadism remains variable and it was reported to be reaching 5.6% according to the Massachusetts Male Aging Study [2].

Despite the fact that the hypothalamus pituitary gonadal axis is affected by different pathologies; idiopathic HH remains the most common cause of HH [3]. HH results from an isolated defect in gonadotropin-releasing neurons with a male-to-female ratio of 3-5:1 and the prototype of HH is Kallmann syndrome which is characterized by anosmia [4].

Infertility is the main consequence of hypogonadism whereas endocrine causes constitute about 15% of infertility etiologies [5]. Male hypogonadism is strongly associated with obesity, metabolic syndrome, and diabetes mellitus (type 2), in addition; persistently unmanaged patient results in many complications such as erectile dysfunction, anemia, osteoporosis, fractures, myopathy, frailty, gynecomastia, psychosocial impairments and reduced quality of life [6].

The diagnosis of HH may be challenging in patients presenting with delayed puberty or lack of secondary sexual characteristics in early adulthood since it is difficult to differentiate HH from a constitutional delay of growth and puberty (CDGP) [4]. CDGP is far more common than idiopathic HH, affecting approximately 3% of adolescents while the incidence of IHH is (one in 30 000) in males [7].

Therapeutic options for treating patients with HH depend mainly on the future desire for fertility. Otherwise, if this is not the point, exogenous testosterone replacement therapy which is capable to induce secondary sexual characteristics is considered the classical treatment option [8]. Human gonadotropin analogs provide direct stimulation of the endogenous testosterone they restore testosterone levels and consequently initiate spermatogenesis [9].

Several protocols have been adopted for treating male patients with HH but there is no general agreement on the optimal option for their management and many protocols depend on expert opinions [10]. The field of male fertility is rapidly advancing as the importance of restoring and maintaining spermatogenesis in men before, during, and after testosterone replacement therapy is becoming fully realized [11].

There is no perfect treatment for HH and most of the available treatments are costly in consideration of the patient’s economic status [12]. However, there is still some uncertainty about the optimal treatment modality, the duration of treatment, and the influence of interfering factors [13]. The study aimed to compare the effectiveness of different gonadotropin modalities in the management of male HH.

## Materials and methods

A randomized open-label prospective study was conducted from June 2021 to December 2022. The total number of patients included was 66 attending Faiha Specialized Diabetes, Endocrine and Metabolism Center (FDEMC) Basrah, Iraq, their main complaint being the lack of secondary sexual characteristics. Patients were considered to have HH based on failure to undergo spontaneous puberty by the age of 18 years, serum testosterone (<100 ng/dL in the setting of low/inappropriately normal gonadotropins (LH, FSH), and no evidence of an underlying cause of hypogonadism.

Fifteen patients were excluded from the study as they failed to attend the follow-up visits or the patients were on testosterone therapy within the last 6 months, patients with other diagnoses such as (hypergonadotropic hypogonadism, Sertoli cell-only syndrome, hemoglobinopathy, any patient with testicular surgery or trauma, pituitary adenoma, apoplexy, Rathke’s cleft cyst, Patients with a history of radiotherapy (cranial) and medications that induced hypogonadism (steroids, opiates, or chemotherapy). All the included patients had informed consent to participate in this study.

Every patient was subjected to a thorough history and clinical examination including a family history of delayed puberty, history of testicular trauma, Mumps in childhood, past history of cryptorchidism and orchidopexy, history of head injury, and drug history including prior testosterone use. An anthropometric examination was done and examination regarding midline defects, the presence of anosmia, gynecomastia, the extent of pubic hair, the presence of eunuchoidism, micropenis, cryptorchidism and mean testicular volume measurement using Prader’s orchidometer.

Baseline testicular volume was measured on the first visit together with, serum testosterone, luteinizing hormone (LH), follicular stimulating hormone (FSH), and estradiol(E2). We measured testicular volume every three months, while measurement of serum testosterone level was done, the seminal fluid analysis also was for those who normalized their serum testosterone at least for three consecutive months.

We randomly divided the patients into three groups according to the treatment mode they received into: The first group was treated with human chorionic gonadotropin (hCG) alone. The second group with a combination of hCG hormone and human menopausal gonadotropin (HMG). While the third group start their treatment with hCG alone for six months and then shifted to a combination of hCG and HMG until the end of the study.

The dose of Recombinant hCG (ovitrelle®) used was 6,500 IU injection subcutaneously every week divided into three doses while HMG (menotropin) dose was 75 IU administered intramuscularly three times weekly. We adopt successful spermatogenesis as the observation of one sperm by microscope in seminal fluid [14]. For each patient, 10 mL of peripheral venous blood was used after being directly centrifuged and analyzed using (Roche-cobas®e411) platform for the measurement of serum testosterone, LH, FSH, and estradiol.

Data were analyzed using SPSS Inc. (Chicago, IL, USA) version 26. Continuous variables including (age, BMI, testicular volume, serum total testosterone, LH, FSH, and E2) were summarized in mean ± standard error of the mean (SEM), while categorical variables including (anosmia, eunuchoidism, cryptorchidism, and past history of testosterone treatment) where represented as a number (percent). The normality of the data was tested by using the Kolmogorov-Smirnov test. We use Student’s t-test to compare means in normally distributed data and the Mann-Whitney U test for non-normally distributed data. Chi-squared test and Fisher’s exact test were used when appropriate for the association between categorical variables, a p-value of < 0.050 was considered statistically significant.

## Results

In Table 1, the baseline general characteristics of the patients at the beginning of the study, where there was no significant difference in the means of age, BMI, serum total testosterone, LH, FSH, and E2 between the three groups, and also there was no statistically significant difference regarding the presence of eunuchoidism, anosmia, cryptorchidism, testicular volume, and previous history of testosterone therapy.

**Table 1:** Baseline characteristics, N=51 Data are expressed either as mean ± SEM or No. (%) Abbreviations: BMI=body mass index, hCG=human chorionic gonadotropin, LH=luteinizing hormone, HMG=human menopausal gonadotropin, combination= (hCG and HMG), sequential= (hCG then a combination of both hCG and HMG), TV=testicular volume, E2=estradiol, FSH=follicular stimulating hormone

	hCG alone, N=16	Combination, N=12	Sequential, N=23	P-value
Age (year)	24.1±1.7	28.2±1.6	26.5±1.2	0.201
BMI (kg/m^2^)	27.3±2.0	27.6±1.8	25.0±1.5	0.506
TV (mL)	5.9±0.6	5.9±1.0	4.8±0.6	0.397
Anosmia	1(6.3)	1(8.3)	2(8.7)	0.959
Eunuchoidism	15(93.8)	11(91.2)	21(91.3)	0.959
Cryptorchidism	2(12.5)	3(25.0)	3(13.0)	0.597
Total testosterone (ng/dL)	14.6±2.7	24.8±6.9	20.8±5.7	0.490
Estradiol (pg/mL)	11.6±2.8	11.8±3.6	16.34±4.9	0.635
LH (mIU/mL)	0.66±0.25	0.39±0.15	0.34±0.08	0.335
FSH (mIU/mL)	1.09±0.27	0.64±0.13	0.66±0.09	0.127
History of testosterone use	7(43.8)	3(25.0)	5(21.7)	0.309

Regarding testicular volume as a main outcome of treatment (Table 2), it increased significantly from the baseline in the three groups (p-value < 0.001) but no significant difference in the final volume was found between the groups at the end of the study (p-value 0.702).

We found an overall increment in the mean serum total testosterone level, which was statistically significant among all the treatment groups (p-value < 0.001). In comparison between the groups, a higher mean maximum testosterone level (710.4±102.7 ng/dL) was obtained with the combination group (hCG, HMG) followed by the sequential group, with mean maximum testosterone levels (636.0±68.6 ng/dL), a p-value of 0.031.

The number of patients who achieved spermatogenesis was nearly equal in this study across the groups with a numerically higher number of patients achieving spermatogenesis in the sequential group but without statistical significance (p-value = 0.882).

**Table 2:** Main outcomes of gonadotropin treatment of hypogonadal patients Abbreviations: Δ TV=final testicular volume minus initial testicular volume, Δ TT=maximum serum testosterone minus initial testosterone level.

	hCG alone, N=16	Combination, N=12	Sequential, N=23	P-value
Testicular volume (mL)				
Final	9.8±1.3	11.4±1.9	10.2±0.9	0.702
Δ TV	4.0±0.9	5.5±1.2	5.4±0.8	0.589
P-value	<0.001	0.007	<0.001	
Testosterone (ng/dL)				
Maximum	413.6±58.7	710.4±102.7	636.0±68.6	0.031
Δ TT	398.9±59.1	685.8±110.1	615.2±67.5	0.040
P-value	<0.001	<0.001	<0.001	
Frequency of patient-achieved spermatogenesis				
Number of patients	4(25.0)	4(33.3)	7(30.4)	0.882
Maximum sperm count	20.3±11.9	14.3±7.7	24.4±10.6	0.804

The factors affecting total testosterone level can be seen in Table 3. In the patients with BMI, greater than 30 kg/m^2^ the final testosterone level is significantly higher in the combination and sequential groups than the hCG alone group which exhibits a significant response in testosterone level in BMI less than 30 kg/m^2^. Initial testicular volume as a factor affecting total testosterone, we found that in patients with greater than 5 mL there is a statistically significant difference in the total testosterone level in the combination group p-value 0.010, while in those patients with their initial testicular volume less than 5 mL, we found the hCG alone group had statistically significant lower total testosterone. Patients who had been treated for less than 13 months with hCG alone had lower total testosterone levels than those with a longer duration of therapy p-value 0. 021. In comparison between groups, the combination group had a statistically significant higher total testosterone level p-value of 0.008. None of the following studied factors (age, basal total testosterone, and basal E2) had an effect on maximum and final total testosterone.

**Table 3:** Factors affecting final total testosterone level Abbreviations: hCG=human chorionic gonadotropin, BMI=body mass index, TV=testicular volume

		hCG alone, N=16	Combination, N=12	Sequential, N=23	P-value
Age	≥26 years	446.0±99.4	695.8±133.2	642.8±97.7	0.329
	<26 years	223.6±74.5	231.2±133.5	348.1±100.5	0.107
	p-value	0.711	0.518	0.927	
BMI	≥30 kg/m^2^	208.0±37.0	522.4±123.2	522.2±76.3	0.037
	<30 kg/m^2^	507.0±67.0	844.7±136.7	667.6±84.5	0.110
	P-value	0.012	0.126	0.395	
Basal TV size	≥5 mL	538.7±62.3	977.0±164.3	664.5±70.6	0.013
	<5 mL	205.0±44.9	520.0±76.0	614.1±110.9	0.044
	P-value	0.001	0.019	0.725	
Median basal testosterone	≥13.5 ng/dL	347.6±67.6	795.9±151.9	606.7±122.0	0.039
	<13.5 ng/dL	498.4±98.7	561.5±153.5	654.9±84.5	0.526
	P-value	0.213	0.342	0.740	
Median basal estradiol	≥5.7 pg/mL	402.5±92.1	552.3±136.9	606.3±164.4	0.589
	<5.7 pg/mL	523.5±113.1	590.6±93.3	457.2±72.0	0.630
	P-value	0.426	0.818	0.473	
The median duration of Treatment	≥13 months	581.3±85.7	608.0±161.2	691.8±88.6	0.739
	<13 months	312.9±61.2	783.6±136.5	508.6±99.7	0.008
	P-value	0.021	0.452	0.227	

In Table 4, the mean basal testicular volume (8.3±1.3 mL) and final testicular volume (14.3±2.3 mL) positively enhance spermatogenesis in hCG alone group (p-value < 0.050). The means of the following factors (age, BMI, basal total testosterone, basal E2 level, and duration of treatment) had no significant effect on spermatogenesis. All the included patients with cryptorchidism had failed to achieve spermatogenesis. Prior exposure to exogenous testosterone had not any significant influence on sperm production.

**Table 4:** Factors affecting spermatogenesis Abbreviations: a= Fisher's Exact test, A=azoospermia, S=spermatogenesis, E2=estradiol

		hCG alone, N=16	Combination, N=12	Sequential, N=23	P-value
Patients achieved spermatogenesis		4	4	7	
Mean age (year)	S	26.3±3.1	27.5±1.9	28.0±2.6	0.903
	A	23.3±2.0	28.5±2.3	25.8±1.4	0.195
	P-value	0.294	0.798	0.592	
Mean BMI (kg/m^2^)	S	29.8±1.4	25.5±2.0	25.5±3.2	0.545
	A	26.3±2.6	28.6±2.4	24.8±1.7	0.512
	P-value	0.394	0.496	0.973	
Mean basal TV size (mL)	S	8.3±1.3	6.5±1.4	5.7±1.3	0.461
	A	5.1±0.6	5.3±1.2	4.4±0.6	0.704
	P-value	0.015	0.298	0.476	
Mean final TV (mL)	S	14.3±2.3	14.3±3.9	12.0±1.7	0.734
	A	8.2±1.2	10.0±2.0	9.4±1.1	0.667
	P-value	0.026	0.198	0.164	
Mean Δ TV (mL)	S	6.0±2.0	7.3±3.6	6.3±1.7	0.936
	A	3.2±0.9	4.6±2.2	5.0±0.9	0.543
	P-value	0.131	0.549	0.501	
Cryptorchidism	S	0	0	0	NA
	A	2(100.0)	3(100.0)	3(100.0)	
Non-cryptorchidism	S	4(28.6)	4(44.4)	7(35.0)	0.738
	A	10(71.4)	5(55.6)	13(65.0)	
	P-value	0.550a	0.255a	0.316a	
Mean basal total testosterone (ng/dL)	S	20.3±7.1	32.5±16.1	16.9±5.1	0.477
	A	12.8±2.7	20.4±6.4	22.5±8.0	0.557
	P-value	0.299	0.450	0.841	
Mean basal E2 (pg/mL)	S	11.5±2.9	15.6±8.5	34.5±28.8	0.424
	A	11.6±4.1	9.9±3.9	13.3±3.9	0.849
	P-value	0.650	0.787	0.254	
History of testosterone use	S	2(28.6)	1(33.3)	2(40.0)	0.918
	A	5(71.4)	2(66.7)	3(60.0)	
No history of testosterone use	S	2(22.2)	3(33.3)	5(27.8)	0.871
	A	7(77.8)	6(66.7)	13(72.2)	
	P-value	0.608a	0.745a	0.492a	
Mean duration of treatment (months)	S	9.0±3.2	14.3±1.6	17.3±1.9	0.070
	A	12.0±1.9	13.1±4.2	16.3±2.0	0.405
	P-value	0.361	0.061	0.383	

## Discussion

The therapeutic approach to managing patients with HH remains variable and depends solely on the age of presentation and the need for future fertility, usually, we receive patients with HH late in adulthood with their main complaint being the lack of secondary sexual features or the concern of fertility [15]. Testosterone therapy was formerly used to manage hypogonadism it induces secondary sexual characteristics and promotes more rapid virilization but it does not induce testicular enlargement or spermatogenesis [8,15].

Congenital HH is considered a rare treatable cause of infertility. Induction of puberty can be achieved with gonadotropin therapy [16,17]. They indirectly stimulate spermatogenesis and induce fertility by increasing endogenous testosterone to higher levels and promoting better physical conditioning [18]. HH patients with anosmia were considered congenital in their nature, and because of limited resources regarding the genetic study, we considered our cases in this study as idiopathic in their origin.

In daily clinical practice patients in whom testicular size is post-pubertal more than 4mL, the induction of spermatogenesis can be obtained by hCG alone [19-22] and if the sperm doesn’t appear in the ejaculate after six months with adequate testosterone level FSH can be added [23], in this study, because most of our patients had testicular size equal or greater than 4 mL which reflect they might be incompletely treated by gonadotropins the transition group treated first with hCG alone then translocated to a combination of LH and FSH for the rest of the study after six months of sustain LH therapy.

Furthermore, when the initial testicular volume is below 4 mL the classical treatment option remains either the GnRH pump, which is not available in our country, or the combination of gonadotropins. In this study, the combination was done randomly and contrary to the common belief we start with LH alone then FSH was added to the regimen rather than LH followed by FSH aiming to reduce the cost because the sperm may appear in the ejaculate with the use of LH alone modality.

Although the patients who were previously treated with testosterone therapy were not excluded due to the reversible effect and restoration of spermatogenesis after testosterone stoppage in more than 90% of the studied patients within six months, we exclude cases with previous testosterone exposure in the last preceding 6 months for recruitment bias [11]. Normal fertility in males depends on the complex and coordinated secretion of GnRH from the hypothalamus and the consequent secretion of gonadotropins from the pituitary gland. The pulsatile nature of this secretion is critical for the testicular response and spermatogenesis [24,25].

The observed fluctuating total testosterone level in this study belongs to the short half-life of hCG treatment and wean off the dose-effect before the next visit but this fluctuation does not result in a fall in serum testosterone below the satisfactory level for spermatogenesis, and we used the difference between maximally recorded testosterone and basal testosterone level (Δ TT). In spite of all groups having a significant increase in both (testicular volume and total testosterone level) and these changes were higher in the patients who were treated with the combination group, these changes may be due to the combination group being more identical to normal physiology and a lower response in the other two groups which may be explained by genetic mutations that affect the response to hCG treatment [26].

In a study done in west China by Yang et al., they conclude that the testicular size increment was more in the combination group at 75% of the total patient number compared with hCG alone at 50%, but; those patients followed up to five years which is parallel to our results [27]. All the patients in this study had an initial testicular volume of more than 4 mL with little difference between the three groups which is thought to be responsible for the final differences in the duration of the treatment needed to get the normal testicular size and spermatogenesis with normal sperm count, these final results may belong to these gonads might be exposed partially to low levels of endogenous gonadotropins or previously treated with HMG; whereas, researchers defined male partial HH patients as those with a testicular volume of ≥4 mL. [14].

Chen et al. study show no difference between hCG alone and the combination group although both groups had significant differences from the baseline testicular volume and these findings are similar to the results shown in Table 2 [26]. The BMI is inversely related to the testosterone level response which is more significant in obese patients and may be due to aromatization and low serum total testosterone in those patients [28].

Obese men also possess heavy adipose tissue depot, which homes several toxins, adipokines, and other hormones (adiponectin, leptin, ghrelin, orexin, obestatin, etc.). High adipose tissue accumulation also leads to increased scrotal temperature, sleep apnea, and systemic inflammation [29]. Finally, as discussed above the total testosterone level will be better in patients whose TV is equal to or more than 5 mL, more than 13 months of therapy duration, and not obese patients and especially in hCG-alone treated patients.

The rate of spermatogenesis in all patients with HH was (64%-75%) in other studies which is higher than our study this belongs to the duration of the study, which limits the final results of spermatogenesis and sperm count and it is recommended to extend the study to two years to get the final optimal results. In addition, some studies mentioned that during the follow-up, 50% of the patients with HH produced their first sperm within (12-18) months of the gonadotropin treatment [27].

All the patients who had cryptorchidism in this study remained had azoospermia during the period of the study in all modalities of therapy but the difference between groups was nonsignificant and this looks similar to the result of Hadziselimovic et al. study where cryptorchidism is an ominous sign and this may be attributed the absence of Ad spermatogonia, which is an important prognostic parameter for future fertility. Cryptorchid boys lacking these cells will develop infertility despite successful orchidopexy at an early age plus gonadotropins replacement therapy [30].

The study has limitations. The unavailability of the GnRH pump which is regarded as the method of choice for replacement therapy as it employs gonadotropin in a more physiological pattern constitutes a major limitation of the study. The time of the study is limited and we expect substantial patients’ number who achieve early sperm appearance after an extended time this study needs to be extended. The seminal fluid analysis was done in different laboratories by different methods.

## Conclusions

Induction of puberty using recombinant hCG alone is sufficient to induce secondary sexual characteristics, while for fertility issues combination from the start or sequential therapy has a better outcome for spermatogenesis. Factors negatively affecting final testosterone level include obesity, lower initial testicular volume, and shorter duration of therapy. There is no effect of prior use of exogenous testosterone on final spermatogenesis. Further studies are needed.
