# Correction: Management Outcomes in Males With Hypogonadotropic Hypogonadism Treated With Gonadotropins

**DOI:** 10.7759/cureus.c290

**Published:** 2025-09-11

**Authors:** Bahaa O Sahib, Ibrahim H Hussein, Nassar T Alibrahim, Abbas A Mansour

**Affiliations:** 1 Diabetes and Endocrinology, Fiaha Specialized Diabetes, Endocrine and Metabolism Center (FDEMC) University of Basrah, Basrah, IRQ; 2 Endocrinology, Faiha Specialized Diabetes, Endocrine and Metabolism Center (FDEMC) College of Medicine, University of Basrah, Basrah, IRQ; 3 Diabetes and Endocrinology, Faiha Specialized Diabetes, Endocrine and Metabolism Center (FDEMC) College of Medicine, University of Basrah, Basrah, IRQ

The Ethics Statement of this article has been corrected to state that this study did involve human subjects and was reviewed and approved by the ethical committee of Faiha Specialized Diabetes Endocrine and Metabolism Center (FDEMC).

